# Prospects and Challenges of Microwave-Combined Technology for Biodiesel and Biolubricant Production through a Transesterification: A Review

**DOI:** 10.3390/molecules26040788

**Published:** 2021-02-03

**Authors:** Nur Atiqah Mohamad Aziz, Robiah Yunus, Dina Kania, Hamidah Abd Hamid

**Affiliations:** 1 Department of Chemical and Environmental Engineering, Faculty of Engineering, University Putra Malaysia, Serdang 43400 UPM, Malaysia; nuratiqah.aziz@gmail.com; 2 Institute of Plantation Studies, University Putra Malaysia, Serdang 43400 UPM, Malaysia; dinakania@gmail.com (D.K.); hamidah1310@gmail.com (H.A.H.)

**Keywords:** microwave, transesterification, biodiesel, biolubricant, combined technology

## Abstract

Biodiesels and biolubricants are synthetic esters produced mainly via a transesterification of other esters from bio-based resources, such as plant-based oils or animal fats. Microwave heating has been used to enhance transesterification reaction by converting an electrical energy into a radiation, becoming part of the internal energy acquired by reactant molecules. This method leads to major energy savings and reduces the reaction time by at least 60% compared to a conventional heating via conduction and convection. However, the application of microwave heating technology alone still suffers from non-homogeneous electromagnetic field distribution, thermally unstable rising temperatures, and insufficient depth of microwave penetration, which reduces the mass transfer efficiency. The strategy of integrating multiple technologies for biodiesel and biolubricant production has gained a great deal of interest in applied chemistry. This review presents an advanced transesterification process that combines microwave heating with other technologies, namely an acoustic cavitation, a vacuum, ionic solvent, and a supercritical/subcritical approach to solve the limitations of the stand-alone microwave-assisted transesterification. The combined technologies allow for the improvement in the overall product yield and energy efficiency. This review provides insights into the broader prospects of microwave heating in the production of bio-based products.

## 1. Introduction

Microwaves have been used in various fields, such as food, chemical, medical, and telecommunication industries. Previous studies have also demonstrated the potential of non-thermal aspects of using microwaves in biological, hydrothermal depolymerization, or carbonization processes [1,2,3,4]. For biological systems, non-thermal effects occur as cell metabolism changes caused by resonance absorption and induced electromagnetic fields, and are often accompanied by a specific behavioral response when neural structures are involved [4]. The microwave is generated via microwave tubes called magnetrons, with high efficiency (>70%), and power output of more than 1 kW [5]. On the other hand, the transformation of thermal energy from electrical in high-frequency microwave assistance generally requires a substance with an asymmetrical molecular structure. For a domestic microwave with a frequency of 2450 MHz, rapid changes of electrical field direction occur 2450 million times in a second. As a result, rapid oscillations of dipole molecules and free ions in the substance will generate an enormous amount of thermal energy, which is then absorbed by the substance. Fast-moving fields are called an electromagnetic waves or electromagnetic radiation, which represent the energy emitted by the substance due to changes in the electronic configuration of atoms or molecules [6,7].

Microwave heating has shown potential applications in biodiesel production through a transesterification reaction [8], syngas production via pyrolysis [9,10,11], food drying [12,13], plastic-polymer synthesis [12], composite reinforcement [14], extraction [15,16], and biogas production [17,18,19]. Microwave irradiation is used to intensify the rate of heating of any process faster than the conventional heating [20]. Microwaves derive heat from the emission of electromagnetic waves of charged proton and electron movements [21]. A reaction mixture that is heated using the microwave is influenced by the interaction between the electric field and the substance. Microwave irradiation penetrates the substance at molecular level and transfers the energy generated within the molecules without necessity to heat the entire reactor [22]. Therefore, a short residence time, high product conversion and yield, low by-product throughput, and efficient energy could be achieved [16,23].

**Figure 1:**
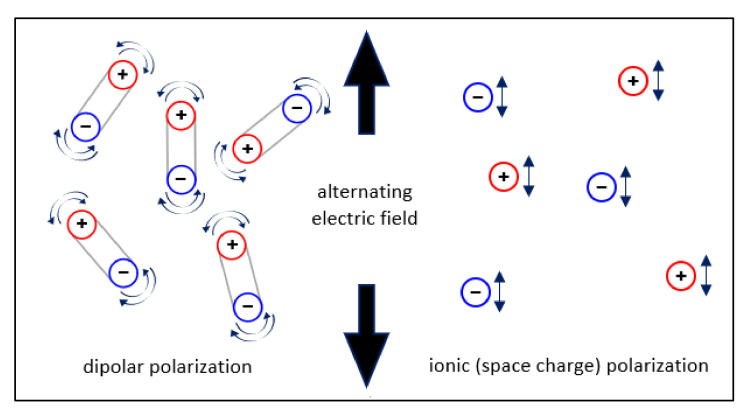
Molecular and ionic movements under microwave influence.

The application of microwave heating requires an understanding of the dielectric properties of the microwave reaction materials [24]. The molecular properties of the substance measure the ability to inhibit the movement of electrons and, thus, create polarizations within the substance when exposed to an external electric field. For lubricating oil, the dielectric properties are among the inherent properties that could be used to assess their purity. Any changes in dielectric constants may indicate the contamination of oil due to the presence of water or changes in oil chemistry due to oxidation or weakening of the additive [25,26]. In a reaction, the dielectric properties of a solvent or material significantly affects the efficiency of microwave heating, while the dielectric properties depend on frequency and temperature [27]. A microwave irradiation generates heat via two main mechanisms, which are dipolar and ion polarizations. The polar molecules in the reaction mixture (e.g., polar-solvent molecules) are involved in the dipole–dipole interactions, while the charged particles in a sample (free ions) are affected by the ionic polarization (see Figure 1). At microwave frequencies, the dipole molecules or ions of the sample are aligned in the applied electric field. Consequently, as the applied field oscillates, the dipole or ion field attempts to re-align itself with the alternating electric field, which results in the energy loss in a form of heat through molecular friction and dielectric loss [28]. 

The microwave heating requires significantly less energy because of localized heating rather than the use of conventional heating through a conduction and convection. The conventional heating depends on the temperature gradient between the internal reactor wall and the reaction mixture, and the conductivity of the reactor wall. The heating of the reaction mixture is established only when the conductive heat transfer through the wall has occurred, after which the heat is transferred to the reaction media by convection. Factors affecting conduction heating are the properties of the material, the temperature gradient, and the cross-section of the reactor and its thickness. The convection heating starts when the fluid near the wall surface is hot. The molecules then gain energy in the form of internal energy as well as from heat of reaction. When the moving fluid is energized (heated), it expands and the density decreases, which causes it to rise and the process continues until the thermal equilibrium is reached. The ability of the microwave to reduce processing time, save energy and improve production yield is attributed to the intense heating at molecular level [29]. The major difference between conventional and microwave heating is shown in Table 1.

Significant developments in biodiesel and biolubricant synthesis involving the raw materials, catalysts, an optimization of operating conditions, have been underway over the decades. The main objective is to optimize the yield of the product at the lowest overall production cost. The process of transesterification in biodiesel synthesis involves the chemical fragmentation of triglycerides into smaller, straight-chain alkyl ester molecules. Glycerol and fatty acid ester molecules are produced by the reaction of one mole of triglycerides with three molar equivalents of methanol. This reversible transesterification reaction is also known as an alcoholysis or methanolysis. Biolubricant, especially polyol ester, on the other hand, is produced by transesterification of fatty acids or fatty acid esters with polyhydric alcohol.

Biolubricant is a safer candidate to mitigate environmental issues contributed by the industrial lubricants in the event of accidental spillage or irresponsible disposal in water or soil [30]. It is a petroleum-free alternative to conventional lubricants due to the depletion of fossil fuels supply. The thermal heating is governed by the process of conduction and convection, while the microwave heating is performed by the electromagnetic wave radiation. In comparison, microwave heating uses less energy compared to thermal heating and reduces the reaction time considerably. To date, microwave heating has started to be the common method of biodiesel synthesis via esterification or transesterification [8,20,31]. Side reactions such as hydrolysis and saponification always interfere with the main esterification or transesterification reactions via conventional heating. Apart from the reduction in product yield, the separation of the product has become more difficult [32]. Microwave-assisted reactions take shorter time and therefore have a potential to mitigate the chances of side reactions occurring.

Alkaline catalysts tend to form metal soap during transesterification because of free fatty acids reacting with the metal component of the catalyst. Hence, saponification reactions can affect product separation and conversion [33]. Regardless of saponification problems, alkaline catalysts still provide greater selectivity for the conversion of plant lipids into methanol than acid catalysts [34]. Moreover, the reaction conversion yields are much lower using the acid catalyst, which requires much more time. The use of small amounts of alkaline catalyst (less than 1 wt.%) has reported to significantly increase product yields [35,36] and it is an attractive option for industry [37,38]. Aside from a longer reaction time, transesterification reaction using an acid catalyst requires higher temperatures compared to an alkaline reaction. Kamil et al. [39] reported that a conversion of 91.5% of jatropha methyl ester at 210 °C at 5 h using sulfuric acid was achieved. In another study, by using Stannous chloride catalyst, 92% conversion of 10-undecenoic acid-based polyol was obtained at 150 °C at 6 h [40]. Other studies also proved that when the transesterification was performed using a microwave heating, the reaction time was only from 10 to 30 min at temperatures below 100 °C [41,42]. The reported fatty acid methyl ester (FAME) conversion yields obtained by using sulfuric and heteropoly acids were 86.7% [41] and 96.2% [42], respectively. A microwave-assisted esterification reaction using sulfuric acid as a catalyst disclosed 99.5% of FAME conversion in 15 min [31]. The transesterification reaction using an enzyme is usually conducted under milder operation conditions because some enzymes denature at higher temperatures. Although high conversion of a desired ester could be obtained, the reaction by using enzymes usually take a significantly longer time than the reaction by using alkaline catalysts [43].

This paper reviews developments in the use of combined microwave techniques in biodiesel production, as well as reviews the present state of technology and provides insights into potential technologies. The literature analysis suggests that several review papers have been published regarding microwave-assisted heating combined with acoustic cavitation, vacuum pump, and supercritical/ subcritical approach to improve chemical reactions, but a comprehensive review of the applications of these combined microwave technologies for biolubricant and biodiesel production is still lacking. This review provides guidance on how the combination of microwave and other techniques can be incorporated into the sustainable production of biodiesel and biolubricants.

## 2. Recent Stand-Alone Microwave Applications

Currently, microwave irradiation is more common in the production of biodiesel than in the productions of biolubricants. Table 2 presents previous stand-alone microwave-assisted transesterification studies with feedstock consisting of methanol or ethanol and various plant oils. In biodiesel production, most researchers conducted experiments at temperatures between 50 °C and 70 °C due to the boiling point of methanol at a temperature of 68 °C [44,45,46,47]. The optimum temperature provides enough energy for the molecules of the reactants to achieve an effective collision. In addition, higher temperatures provide higher solubility of the reactants, which contributes to higher product conversion [33]. Methanol is used in excess simply to push the forward reaction and produce more methyl ester. Otherwise, a longer reaction time is needed to complete the reaction. Studies have found that the molar ratio of oil to methanol used by researchers was as low as 1:6 and up to 1:60 with a conversion of 71.4 to 98.9%, as shown in Table 2. For high viscosity oils, a large amount of methanol would be required to enhance the oil solubility in methanol [33].

An improved process for the transesterification of biodiesel fuels and fuel additives involving the use of microwave energy had been patented [53]. It was claimed that the improvements comprise of directing radio frequency microwave energy to reduce an amount of unreacted alcohol and glycerin retained in the biodiesel product and to reduce a time required for separating the biodiesel product from the unreacted alcohol and glycerin. Similar results by using different feedstocks in the microwave reactor were claimed by other patents [54,55]. The scaling up potential of microwave-assisted reactions has been thoroughly reviewed by La Hoz et al. (2011) [56], which reported that a microwave reactor up to 12 L was studied in organic chemistry reaction. In 2017, the use of microwave-assisted transesterification for biodiesel was still undergoing laboratory-scale experiments [57]. To date, its application has yet to be implemented at the industry level. Most microwave assisted reactions in alcoholysis of triglycerides reaction that have been reported at the laboratory scale are for reaction volumes up to 80 g in 250 mL reactor [58], and reaction times of less than 20 min [46,58]. Furthermore, Gude et al. [59] explain that batch process would be not efficient when conducted at a larger scale. Hence, the reacting materials that are continuously pumped and heated in a microwave cavity would be more suitable. However, at the current state, an efficient process for a continuous flow has yet been established, which takes into account of an addition of momentum transfer to the heat generation from microwave heat transfer in the solvent/solid matrix, mass transfer through the solid/solvent [60].

## 3. Limitations of Stand-Alone Microwave Technology

Although a great deal of research has been done on the microwave-assisted synthesis of biodiesel and biolubricants, some issues related to microwaves, such as arching [61], overheating, and explosion [62] still exist and need to be addressed. Measuring the inside temperature of the reactor during the reaction in the microwave oven may be a challenge for the k-type thermocouple. If there is interaction between the metallic probe and the microwave, the presence of the thermocouple inside the cavity could create an arc [63]. Arc or spark may also be present in the microwave due to the metal scarp and chipped paint on the inside surface. Alternative devices, such as infrared thermocouple and fiber optic may not be preferred since infrared thermometer is not accurate; only the surface temperature is measured. On the other hand, the fiber optic is expensive and unable to measure sample block temperature [63]. In the pyrolysis process, for example, arc occurs intermittently when carbon beds, waste oil, and metallic char are present in mixtures and temperatures above 600 °C are reached.

The lack of uniformity in a temperature distribution due to the limitation of microwave penetration depth had also been addressed [63]. Other research further found that the local overheat problems and hot spots could lead to thermal leakage, such as sudden system temperature surges [64]. Hot spot or uneven heating occurs with materials whose dielectric loss increases with temperature. In addition to these factors, a lack of data on dielectric properties over a range of temperatures and the microwave frequency that cannot heat transparent materials also contributed to the limitation of microwave ovens [7]. As a result, yield and selectivity of transesterification product would be affected [61,64]. It can also produce arc and sparks when exposed to radiation and electric field at high vacuum conditions in aviation industries. High-energy electrons that have the potential to affect the metallic structure therefore deposit enough energy to produce unwanted secondary electron emissions. Metaxas (1991) understood that pressure should be maintained at around 100–200 mmHg (133–266 mbar) to avoid the formation of microwave plasma under vacuum conditions.

Three other limitations of microwave technology, including scalability, limited application, and health hazard have been addressed by other study [65]. The wavelength of a microwave is short (12.2 cm); hence, it is a crucial criterion to consider a reactor scale-up. Microwave-assisted reactions have a high cost of production compared to most chemical reactions, particularly in the production of biodiesel. However, the microwave’s dependence on the dielectric properties of the substance may result in a low product yield for non-polar reactants. In addition, a low-frequency range of microwave has a high wavelength and can penetrate the human skin, while a high-frequency range may target certain organs in the body. Prolonged exposure of the body to high-frequency microwave may result in damage to DNA strands, body tissues and cells [65].

Microwave heating is a convenient process for drying polar compounds. The presence of water draws the -OH bond to align and continuously change the magnetic field. This motion leads to molecular friction, leading to heat generation. Heating non-polar molecules by microwave is not effective and therefore requires some modification, such as the addition of polar substances to the reaction mixture. Previous studies have investigated the effects of adding both passive heating element and ionic liquid to heat non-polar substances (toluene, chloroform, and hexane) [27,66]. Sintered silicon carbide (SiC) cylinders were used to improve heating of organic substances, such as in Claisen re-arrangement, Diels-Alder reaction, Michael Addition, *N*-alkylations, and Dimroth rearrangements [67]. Carbon-based catalyst is another microwave absorber that has been used in various processes, particularly pyrolysis and syngas reforming processes. Moreover, a metallic char has a high electrical discharge characteristic, due to an oscillating electromagnetic field, thus would enhance the microwave-assisted reaction to a greater extent [61]. Furthermore, previous study revealed that the char had effectively absorbed the microwave due to the selective heating mechanism with higher conversion (82%) compared to the conventional method (65%) [68]. The limitations of stand-alone microwave reactor have been summarized in Table 3.

## 4. Intensification of Microwave-Combined Reactor Technology

### 4.1. Simultaneous Microwave and Acoustic Cavitation Technology

The transesterifications of biodiesel and biolubricant involve multiphase reaction mixtures, such as the mixture of oil-alcohol for biodiesel synthesis and ester-alcohol for biolubricant synthesis. A chemical process intensification is crucial to improve an emulsification of two immiscible liquids in the reaction media. Moreover, a formation of sufficiently small droplet size of reaction mixture would result in the increase of contact surface area between reactants and thus, intensify the transesterification process. Recently, a combination of two simultaneous reactor technology involving microwave has been explored to enhance the mass and heat transfers and at the same time could reduce the energy consumption during the transesterification reaction.

An acoustic cavitation has been widely used to produce biodiesel and accelerate the transesterification reactions with either homogeneous or heterogeneous catalysts [69,70,71]. This technology refers to the process of applying sound energy with frequencies of pressure oscillation above 20 kHz [72]. This method is capable to promote homogenization between the reagents through acoustic cavitation, thus increase the conversion of esters at reduced reaction times, with lower production costs [73]. Acoustic cavitation means the formation and collapse of bubbles in liquid irradiated by extreme ultrasound, often exceeding the sound velocity in the liquid [74]. The bubble collapse is a quasi-adiabatic mechanism in which there is significant thermal conduction between the bubble’s interior and the surrounding liquid. A bubble thus releases a shock wave into the surrounding liquid just after the end of the collapse of the bubble [74,75]. Hence, an apparent superiority of ultrasound technology compared to conventional heating is the generation and collapse of bubbles.

**Figure 2:**
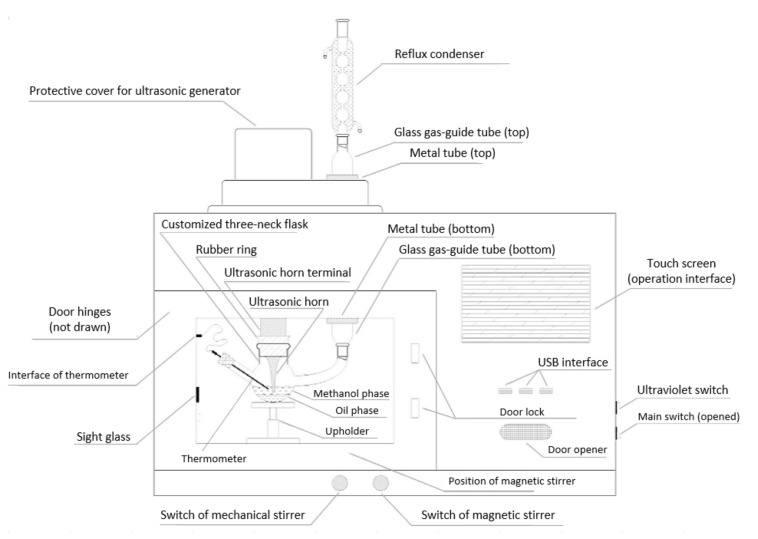
Synergistic microwave-ultrasonic reactor for transesterification of soybean oil with methanol. Reproduced with permission from *Ultrason. Sonochem*. **2017**, 281–290. Copyright (2017) Elsevier [69].

The combination of microwave with either ultrasonic cavitation or hydrodynamic cavitation (see Figure 2) would reduce the mass transfer limitation of a material miscibility during the transesterification [71]. The accumulation of high local energy is generated by ultrasonic cavitation due to the formation and disruption of micro bubbles in the shock wave. Thus, intense density and rapid turbulence flow of liquid, hot spot, and free radical are developed. The hydrodynamic cavitation, on the other hand, uses a Bernoulli principle, which states that velocity of fluid increases inversely with pressure. Bubbles or cavities are formed when the liquid begins to pass through a constriction due to an abrupt increase in the fluid velocity and a decrease in the liquid pressure below the vapor pressure. When the pressure expands, causing the pressure to fluctuate, the bubbles, and cavities collapse downstream. This rapid collapse generates enormous energy and results in the local heating [71].

A combination of microwave and ultrasound technologies in sequential or simultaneous mode has been studied for biodiesel production. Gole and Gogate [76] developed a new approach for the intensification of biodiesel synthesis from high acid value feedstock using a two-step method: microwave sequential effect followed by ultrasound. A large degree of process intensification was caused by synergy between the physical effect of micro-emulsification, the acoustic streaming of ultrasound, rapid heating and the dipolar rotation of microwaves gives. Based on optimization studies, the benefits of sequential effect were reduced reaction times and the need for a smaller amount of excess methanol for equivalent equilibrium levels, which can lead to considerable energy savings in downstream separations.

Ardebili et al. [77] conducted a simultaneous ultrasound-microwave irradiation to produce biodiesel. This technique could significantly accelerate the palm oil transesterification reaction with the optimum parameter as follows: 136 and 129 s sonication and microwave irradiation times, respectively, 1.1% catalyst concentration and a 7:3.1 methanol/oil molar ratio at 58.4 °C to obtain 97.5% biodiesel yield, as in Table 4. The study also concluded the advantages of integrated flow reactors combining high-shear mixing and microwave irradiation for biodiesel production, which are a reduced reaction time as compared to previous works, where ultrasound and microwaves have been applied in a sequential mode and improved biodiesel yield.

Furthermore, Martinez-Guerra and Gude [78] evaluated the synergistic effect of microwaves and ultrasound on transesterification of used plant-based oils. The study found that an equivalent rate of exposure to microwaves and ultrasounds resulted in higher yields of biodiesel for both methanol and ethanol as a reactant. The molar ratio of 9:1 (alcohol to oil) was optimal for both ethanol and methanol with approximately 98% and 96% of biodiesel yields in 2 min reaction time, respectively. The research study revealed that methanol and ethanol are generally good microwave energy absorbers due to their polarity, but the use of ethanol resulted in higher biodiesel yields due to higher solubility properties of ethanol. The study also suggested combining both alcohols to improve biodiesel yield and quality.

When using a combined microwave-ultrasound technology, operating power and time of the process play significant roles in enhanced transesterification. Martinez-Guerra and Gude [79] suggested that optimum power density must be determined for energy-efficient biodiesel production. In addition, Yu et al. [69] found that by measuring the variation of real-time temperature and microwave power during transesterification reactions with different microwave operation time and comparing the corresponding yield, the acceleration of microwave-assisted transesterification was found to be the polarization and further activation of the reactants caused by microwave-assisted transesterification.

Even though combining microwave and acoustic cavitation technologies appears promising, the use of this method is still new to biodiesel. As for biolubricant production, research studies on the use of combined microwave-ultrasound cavitation reactor are still limited. However, the use of a stand-alone acoustic cavitation for the transesterification of bio-based resources had a better exergy efficiency in the biodiesel production, whereby an energy saving around the hydrodynamic cavitation reactor was better than the use of the ultrasound biodiesel reactor [80]. Patience et al. [81] found that the ultrasound-assisted biolubricant production was significantly fast; approximately 2 h faster than the conventional stirring process with the US 500 and 750 W horns. On the other hand, the transesterification of safflower oil a hydrodynamic cavitation reactor to produce biodiesel resulted in 89.1% yield within only 64 s reaction time. Hence, the application of combined microwave and acoustic cavitation could have a high potential to intensify the transesterification to produce biodiesel and biolubricant.

Although the development study of a combined technology of microwave and hydrodynamic cavitation is still limited, the use stand-alone hydrodynamic cavitation-assisted reactor had resulted in 98.1% conversion of methyl ester from waste cooking oil and methanol in 15 min by introducing 21 of 1 mm diameter holes in the reactor orifice. Furthermore, 96.5% conversion of methyl ester from rubber seed oil was also reported by using the reactor in 20 min reaction time with the reactor capacity of 50 L [82]. Although the hydrodynamic cavitation has been used in the production of biodiesel, this method is more effective for biochemical industries, such as wastewater treatment [71,83].

### 4.2. Simultaneous Microwave and Supercritical/ Subcritical Approach

**Figure 3:**
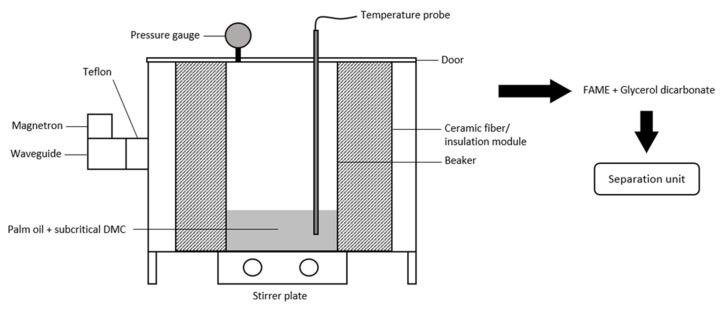
Microwave-assisted transesterification of triglycerides from palm oil with subcritical dimethyl carbonate.

Transesterification of bio-based resources via a supercritical or subcritical approach is catalytic-free, which simplifies the process, it does not require pretreatment or purification steps and is a wastewater-free process [84,85] (see Figure 3). However, both supercritical and subcritical reactions are performed at high temperatures and high pressures requiring high-energy consumption. A common solvent, such as water, was utilized under subcritical conditions; 488 K and 2 MPa for the enhancement of oil extraction of wet *Jatropha curcas L.* (JCL) seed by increasing the porosity of the sample, thus weakening its structure [86]. Furthermore, a lipid hydrolysis became more feasible therefore, transesterification of FAME was achieved at lower activation energy, at a milder condition of 523 K (250 °C) and 13 MPa. In 105 min of an in-situ transesterification reaction, 65.1% yield was obtained by Go et al. [86] using methanol and acetic acid solvents. It took 40–45 min to reach 523 K without microwave heating. Previously, in the non-catalytic FAME production of transesterification of rapeseed oil and methyl acetate in supercritical condition, a molar ratio of rapeseed oil to methyl acetate 1:42, 97% of yield was achieved in 45 min at 320 °C, 20 MPa [87]. By using methyl acetate as a solvent, the triacetin byproduct was proved to increase the oxidative stability and reduces the pour point of biodiesel [87,88].

Further improvement in the process condition by lowering the operating temperature was later reported by introduction of two steps of subcritical acetic acid and oil for fatty acids and triacetin production, followed by using a supercritical methanol for the conversion of FAME in a milder environment. After reaction times in 5 min at 300 °C, 20 MPa and 15 min at 270 °C, 17 MPa, the yield was 96%. This has remarkably shortened the total reaction time [89]. On the other hand, a one-step biodiesel production under supercritical methanol with the presence of acetic acid and CO_2_ at a milder condition [90] had been suggested. The CO_2_ application allowed the reaction to take place at 280 °C, 20 MPa with a yield of 97.8% in 90 min. In addition to the longer reaction time, the ratio of oil to methanol used was as large as 1:60. In addition, Aboelazayem et al. [84] bridged the gap in non-catalytic biodiesel production by obtaining high biodiesel yield using high acidic value waste cooking oil (WCO). By using supercritical methanol with a molar ratio of oil to methanol 1:25, a high biodiesel yield of 98.8% at 265 °C and 110 bar was recorded in 20 min reaction time.

A research study was conducted on a combined microwave-subcritical assisted transesterification of palm oil with dimethyl carbonate (DMC) as a solvent [85]. In 2.5 h, at 167 °C and 5 bar, 86% of the biodiesel yield was obtained with a molar ratio of oil to DMC ratio 1: 9.5. DMC is usually the preferred solvent because it produces a higher value byproduct i.e., glycerol carbonate, which particularly useful in a polymer field. A milder operating condition may take longer reaction time, but there is more energy savings and less hazard. A conventional (without microwave) supercritical condition for palm oil-based biodiesel with similar solvent, DMC recorded 97.4% FAME yield at 300 °C, 20 MPa, 1:42 oil to DMC molar ratio at 20 min [91]. While under slightly different operating conditions, such as at 380 °C, 15–25 MPa, 1:39 oil to DMC molar ratio achieved 91% of FAME in 30 min [92].

Some researchers have suggested that polar substances reduce their polarity or dielectric constant at increasing temperatures [88,92,93,94]. Due to the changes in the dielectric properties of methanol or ethanol during reaction to this condition, Patil et al. [94] introduced heat to intensify the heating by using the microwave irradiation. In the single step of transesterification of algae for the production of biodiesel under microwave heating, the condition was set at 265 °C at 80 bar with a total reaction time of 20 min at 1:9 (wt./vol) ratio of ethanol [94]. In high pressure and temperature conditions, the presence of water from wet algal biomass in the system contributed to the solubility of non-polar organic compounds, which further stimulated the reaction. However, the excessive volume of ethanol required in this process could make the approach less attractive. The previous works mentioned was summarized in Table 5. The combination of microwave at supercritical/subcritical condition for transesterification of triglycerides was limited that most previous work presented were without microwave assistance, as tabulated in Table 5. Thus, it may seem that without microwave assistance a higher yield was produced. A rationality for lack of combination approach is understandable because the supercritical/subcritical require a sophisticated reactor that can sustain high pressure and high temperature condition. The materials must also be suitable and safe to be used in the microwave, which limits the exploration.

### 4.3. Vacuum-Microwave Combined Technology

**Figure 4:**
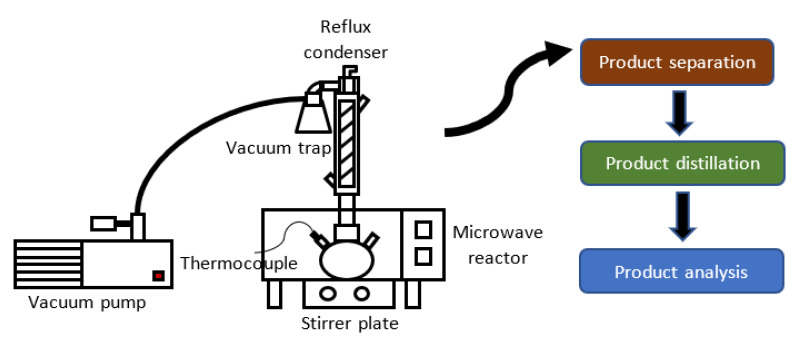
Schematic diagram of vacuum-microwave assisted transesterification reactor system.

In the biodiesel production, vacuum pumps are mainly used in the separation of biodiesel and the by-product, methanol recovery and glycerin purifying. The utilization of vacuum pump can shorten the reaction time as it drives the reaction in the forward direction by removing the volatile by-product under continuous vacuum (see Figure 4). In vacuum condition, the boiling point of the by-product is much lower, making it easier to remove [58,96]. Vacuum treatment can also minimize air resistance and as a result, promote the diffusion of water and reagents into the substance, increasing the contact area so that the reaction is accelerated [97].

The use of vacuum in a conventional heating was reported to increase yields of transesterification and esterification [98]. A vacuum state causes the methanol and oil boiling point to drop to lower than atmospheric conditions. Jookjantra and Wongwuttanasatian [99] produced biodiesel from refined palm oil with heterogenous CaO catalyst in a vacuum reactor assisted by pulse ultrasonic waves. The vacuum condition led to a more productive reaction compared to that under atmospheric conditions. Xie et al. [96] developed a high vacuum distillation method to remove the sulfides by-product in crude biodiesel. The study concluded that high vacuum distillation is an efficient method of eliminating sulfides that are evaporated with light and heavy fractions, producing low sulfur content biodiesel of high quality.

When combined with a microwave, Khalfan and Dwivedi [100] reported that a vacuum pump could help remove excess methanol after a microwave-assisted transesterification of waste cooking oil into biodiesel. A study on a semidry esterification method conducted by Tong et al. [97] also used a microwave-vacuum treatment to increase the reaction efficiency of octenylsuccinic anhydride (OSA) corn starch production. This study showed that this new microwave combined method was able to produce OSA starch with better emulsifying ability, compared to the treatment with the microwave only. The performance relies on the means of microwave radiation to lower the activation energy of the reaction and the ability of a vacuum to increase the internal pressure of starch granules that caused the starch granules to expand, and the structure became fragile to react with reagent more easily. This might be because the initial vacuum phase intensified the transfer phenomena and mechanical effect of abrupt vacuum decompression leading to weakness of starch granules [101]. Overall, this combined technology can accelerate the progress of the reaction.

Aziz et al. [58] evaluated the potential of a vacuum operated microwave reactor for the transesterification of palm oil methyl ester with trimethylolpropane (TMP) biolubricant. A vacuum was installed to produce more TMP triester and help with the removal of the methanol by-product via vacuum suction. The microwave heat was rapidly released within the reaction media as a result from the rotation of molecular dipoles of TMP and methyl ester. The optimum condition was achieved in a short time of 10 min, 63 wt.% of TMP triesters was achieved at 130 °C, 0.6 wt.% sodium methoxide, molar ratio TMP: methyl ester of 1:4, and under 10 mbar vacuum pressure. In comparison, the conventional method took 1 h to achieve similar conversion. An increasing trend of TMP triester composition at increasing temperature was observed. By using the combined microwave and vacuum method, the total energy requirement was markedly reduced by 68.4% compared to the conventional heating method. Moreover, the reaction was 3.1 folds faster in the presence of microwave irradiation.

### 4.4. Simultaneous Microwave and Ionic Solvent Approach

**Figure 5:**
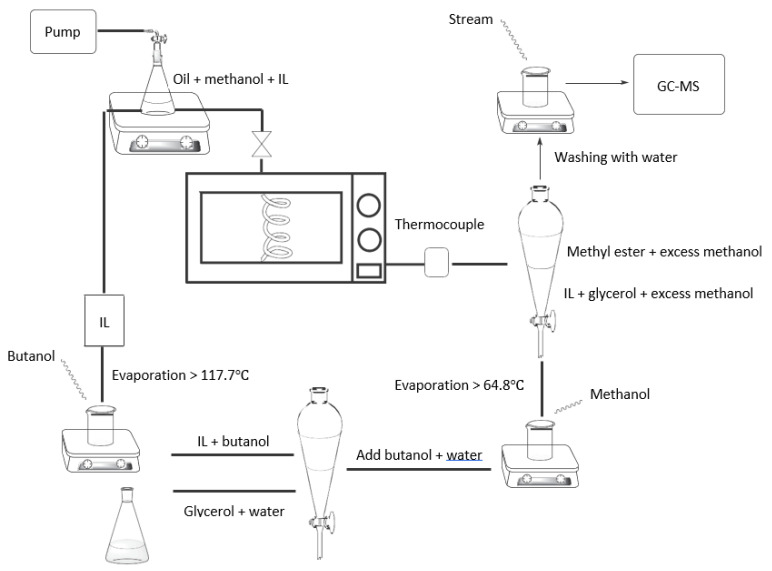
Biodiesel production for microwave and ionic approach. Reproduced with permission from *Ren. Energy* **2020**, 925–936. Copyright (2020) Elsevier [102].

In the microwave-assisted heating method, heat transfer via dipole and ionic polarizations are attractive attributes that contribute to an efficient heating. Microwave heating has benefitted from the polarization of alcohol (methanol and ethanol) and oil or fatty acids in conventional biodiesel and biolubricant production methods. With an addition of sufficient free ions as a heating carrier/passage in a sample, higher microwave absorbance characteristic would be achieved and ionic polarization would be intensified. Researchers are recently explored in-depth for the use of microwave and ionic solvent or ionic liquid (IL) as a catalyst to enhance the thermal effect of microwave heating in biodiesel production (see Figure 5).

Biodiesel production using ionic solvent under microwave heating was summarized in Table 6. This approach highlights the current green solvent, which could also act as a catalyst to drive the reaction forward. Moreover, the solvent or catalyst used can also be recycled and reused [102]. The concept of eutectic solvent is to equip the solvent with appropriate hydrogen bond donor and hydrogen bond acceptor to maximize the interaction between the solvent and substrate for methylation [103]. Control of the ratio of methanol, solvent, and oil is an important key not to over-dilute the mixture or not to provide insufficient acyl acceptor medium. Both conditions would reduce the activity of fatty acids. In addition, the application of microwave increases the coalescence of the fatty acid acyl donor under acidified conditions, thus improving the mass transfer area for interaction of these components. This would lead to a feasibility of the formation of biodiesel due to the enhanced interaction [103].

The comparison of a conventional and microwave heating for the biodiesel production with imidazolium-base IL had been investigated [105]. It took 6.4 h using microwave, while the reaction reached 8 h with a slightly lower yield under conventional heating. The used of imidazolium-based ILs was more well-known to be productive for the extraction and transesterification reaction of algae [103,105,106]. However, Olkiewicz et al. [108] advocates the concern on the imidazolium-based IL price is relatively more expensive than the phosphonium-based IL. The mass production of phosphonium-based IL has managed to control the market price, thereby increasing its accessibility and making it an option for IL. A comparison between imidazolium and phosphonium-based IL in lipid extraction for biodiesel production has shown that phosphonium-based IL is capable of extracting higher lipid percentage, 18.5% and 23.4%, respectively [108]. In addition, added-value byproducts, such as cellulose and proteins, were also obtained. The advantages and disadvantages for overall microwave-combined technology are tabulated in Table 7.

## 5. Prospects and Challenges of Microwave-Combined Technology

The efficiency of combined microwave technologies in the intensification production of biodiesel and biolubricants was recognized by comparing their performance with the conventional and stand-alone microwave heating. The combined technology approach involve undoubtedly new techniques that have a promising potential for applications in the processing of fine chemicals on an industrial scale. Currently, the potential of microwave technology to produce biodiesel and biolubricants are mostly conducted at a laboratory scale using relatively small quantities of raw materials. Nevertheless, previous findings have served as a solid basis for the expansion of the microwave-based transesterification technology to a larger scale. Several studies suggested developing continuous flow and higher capacity system for microwave and other technology enhanced biodiesel production for a more energy-efficient and chemical saving system [77,78]. This is especially important for large-scale commercialization that may need continuous reactor systems, pilot-scale tests, as well as techno-economic feasibility studies.

The use of a combined microwave-acoustic cavitation reactor has high potential to shorten the reaction time significantly, with high yield of products, excellent energy savings, and minimal utilization of hazardous chemicals [77,112]. The challenges of using this combined technology are the difficulties in minimizing its high operating cost and scaling-up for an industrial purpose [113]. Meanwhile, the approach of microwave and ionic liquid or microwave and subcritical/supercritical solvent is an option for a green synthesis with a catalyst elimination. However, to conduct a reaction at subcritical/supercritical condition is energy consuming, while the use of appropriate IL can further reduce the overall production cost and re-use for several times. Microwave technology generally requires more effort in transporting equipment, minimizing dielectric losses, and maximizing the homogeneous distribution of electromagnetic field during the reaction. This technology is therefore suitable for high-quality products, such as biodiesel and biolubricants. The vacuum technology would be attractive for industries because economic analysis has shown that the biodiesel distillation facility can produce revenues of more than $1.8 million per year, suggesting that high vacuum distillation is economically feasible [96]. Meanwhile, research on design and validation of large-scale sonochemical reactors for ultrasonic method has also been undertaken by Gogate et al. [116] to ensure that large-scale development of biodiesel using ultrasonic irradiation could take place in the future. In addition, life cycle assessment (LCA) of a complete system of each approach is essential in the future, including all input materials, energy, production, transportation, and disposal management.

According to the most recent studies, the method of transesterification with microwave hybrid technology for biodiesel and biolubricant production is currently at the laboratory validation level (for technical readiness level (TRL 4)). The production facilities are still working in batch process and not in an entire logistics material chain. The application of microwave hybrid technology is under research and development, similar with various conversion routes such as gasification, hydrodeoxygenation, and pyrolysis. Compared with other biofuel production, such as pyrolysis, the technological maturity of pyrolysis suggests that there is a viable opportunity to further develop this technology as technological developments move towards commercial applications [117]. The challenges for microwave-assisted reactions are the depth of microwave penetration for large batch reactors and the intrinsic temperature gradients for tubular flow reactors [66].

## 6. Conclusions

This review article provides a summary of research findings in the field of microwave-assisted transesterification processes combined with other technologies. This review has demonstrated the advantages of combining microwave heating and other technologies, such as acoustic cavitation, ionic approach, supercritical/subcritical, and vacuum. As compared to the stand-alone microwave assisted reactor technology, the innovative combined technologies with microwave adapted for biodiesel and biolubricant production has great advantages, such as faster heating, lower energy consumption, shorter processing time, higher production yield, and product quality improvement. In a combined microwave-acoustic cavitation technology, acoustic cavitation could provide a mechanical mixing effect needed by microwaves to improve mass and heat transfer, while microwaves generate an excellent thermal energy. Hence, the assistance of microwave-acoustic cavitation during the synthesis of biodiesel or biolubricant clearly takes advantage of the high-energy impact of microwave and acoustic cavitation to address certain shortcomings of conventional processes. On the other hand, combining a microwave with a vacuum pump drives the reaction in the forward direction by removing the volatile by-product under continuous vacuum; therefore, the reaction time can be shortened. The subcritical/supercritical condition with the microwave approach may simplify the separation and purification steps by omitting the catalyst. Adversely, this strategy is correlated with higher solvent demand and energy usage. Microwave-assisted transesterification of triglycerides with ionic liquid combination may somehow benefitted the reusability of the solvent provided. The combination technology could benefit the industry in terms of production time and cost. However, most previous studies are still in laboratory scale and there are still limited scientific reports on the upscaling application of this method to the industrial level, as the technology is relatively new. Detailed research studies on process design, reaction kinetics, thermodynamics, and biodiesel/biolubricant analytical protocols for the larger production are needed for the future up-scaling process for the industrial production of biodiesel and biolubricant. With strict regulations and an increase in the use of renewable energy, microwave-assisted biodiesel and biolubricant synthesis with combined technologies may become more common in the future.

## Figures and Tables

**Table 1:** Comparison of conventional and microwave reactor.

Features	Conventional	Microwave
Reaction time	Longer processing times	Very short, instant heating
Solvent requirement	No or low solvent	No or low solvent
Product quality	Vary	Better
Product yield	Vary	Higher
Separation time	Long time	Very short time

**Table 2:** Stand-alone microwave-assisted transesterification.

Temperature (°C)	Catalyst	Molar Ratio Reactant	Product	Yield (%)	Conversion (%)	Reference
60	1% Sodium methoxide	Oil:methanol (1:6)	Methyl ester	99.7 in 5 min	98.9	[45]
60	1.5% KOH	Oil:ethanol (1:10)	Ethyl ester	80.1 in 10 min	NA	[47]
60	0.5% NaOH and 1% KOH	Oil:methanol (1:6)	Methyl ester	97.5 in 10 min	NA	[48]
60	Heteropoly acid	Yellow horn oil:methanol (1:12)	Methyl ester	96.2 in 10 min	NA	[42]
65	3% Calcium oxide	Soybean oil:methanol (1:7)	Methyl ester	NA	96.6 in 60 min	[49]
70	250 mg Novozym 435	1,2 propanediol:dimethyl carbonate (1:3)	Propylene carbonate	NA	93 in 6 h	[44]
80	4% Tungstated zirconia	Oil:methanol (1:45)	Methyl ester	NA	51.94 in 20 min	[46]
90	3% H_2_SO_4_	Oil:methanol (1:8)	Methyl ester	86.74 in 30 min	NA	[41]
120	1.0% Sodium methoxide	Oil:methanol (1:6)	Methyl ester	NA	96 in 30 s	[50]
170	10% Montmorillonite KSF	Rapeseed oil:methanol (1:18)	Methyl ester	57 in 60 min	NA	[51]
Not mentioned	4.86% Calcium oxide	Nyamplung oil:methanol	Methyl ester	93 in 10 min	NA	[52]

NA: not available.

**Table 3:** Limitations of stand-alone microwave reactor.

Issues	References
Arching	[61,63]
Overheating and explosion	[62]
Expensive fiber optic thermocouple and difficult measurement of block temperature	[63]
Non-uniform heating, hotspot	[7,61,63,64]
Scalability, limited field of applications and health hazard	[65]

**Table 4:** Transesterification via combined microwave-acoustic cavitation technology for biodiesel production.

Material	Operating Conditions	Ester Content	Energy Assessment	Reference
Nagchampa oil Catalyst: Potassium hydroxide	15 min (esterification) and 6 min (transesterification), 100–140 W (MW) 20 kHz 120 W (US) and molar ratio of 1:2	NA	2.5 × 10^2^ kJ/kg	[76]
Waste oils Catalyst: Sodium hydroxide, NaOH	2 min, 300 W (MW-US) and molar ratio of 9:1 (alcohol to oil)	98% biodiesel yield	NA	[78]
Palm oil Catalyst: KOH	2.2 min, molar ratio of 7:3.1 methanol/oil, 58.4 °C	97.5% biodiesel yield	0.36 MJ/L	[77]
Soybean oil Catalyst: KOH	6 min, 65 °C and 700 W (MW) 800 W (US)	98.0% FAME yield	NA	[69]

NA: not available.

**Table 5:** Transesterification of triglycerides from different sources via supercritical/subcritical solvent with or without microwave assistance.

Material	Operating Condition	Ester Content	Energy Assessment	Reference
Palm oil Solvent: dimethyl carbonate	DMC to oil 9.5:1 167 °C 2.5 h, 5 bar	86% yield	Microwave-assisted Ea: 44.88 kJ/mol	[85]
Waste cooking oil Solvent: methanol	Methanol to oil 37:1 253.5 °C, 198.5 bar, 14.8 min	91% yield	Without microwave Ea: 50.5 kJ/mol	[84]
Wet whole kernel Solvent: subcritical methanol and acetic acid	1st step: supercritical water treatment on kernel 488 K, 2 MPa, 15 min 2nd step: subcritical methanol and acetic acid 523 K (250 °C), 13 MPa, 105 min	65.1% yield	Without microwave (2-step approach)	[86]
Algae Solvent: ethanol	1:9 (wt./vol) ratio of ethanol, 265 °C, 80 bar, 20 min	30% yield	Microwave-assisted Heat energy supplied: 1560 kJ Thermal energy associated with sample volume with wet algal: 35.03 kJ	[94]
Microalgae Solvent: methanol, dimethyl carbonate and methyl acetate	Methylating agent to algae 10:1 518 to 643 K, 20 MPa, 10 to 80 min	>90% (methanol) 50% (DMC) 40% (methylacetate)	Without microwave	[95]
Rapeseed oil Solvent: dimethyl carbonate	DMC to oil 42:1 300 °C, 20 MPa, 20 min	97.4% yield	Without microwave	[91]
Soybean oil Solvent: supercritical methanol, carbon dioxide and acetic acid	Methanol to oil 60: 1, acetic acid to oil 3:1 280 °C, 20 MPa, 90 min	97.8% yield	Without microwave	[90]
Rapeseed oil Solvent: subcritical acetic acid and supercritical methanol	1st step: acetic acid to oil 54:1 300 °C, 20 MPa, 30 min treatment 2nd step: reactant to methanol 1:1.6 270 °C, 17 MPa, 15 min	97% yield	Without microwave (2-step approach)	[89]
Palm oil Solvent: dimethyl carbonate	DMC to oil 39:1 380 °C, 20 MPa, 30 min	91% yield	Without microwave	[92]

**Table 6:** Summary for microwave and ionic solvent approach.

Material	Operating Condition	Ester Content	Energy Assessment	Reference
Oleic acid and methanol esterification Catalyst: 1-(4-sulfobutyil)-3-methylmidazolium hydrosulfate, [Bsmim]H_2_SO_4_	10–20 min 100–140 W (MW) 40 kHz 50 W (US) MR of M:O 20–24 Mass ratio IL:O 8–12	97. 85% of oleic acid conversion (17 min, 118 W, M:O 22, mass of IL:O 11)	1.75 × 10^4^ kJ/kg	[104]
Palm oil and methanol Catalyst: Choline hydroxide (ChOH)	400–800 W ChOH 2–6% (*w*/*w*) M:O 9:1–15:1	90% of biodiesel yield, 800 W, M:O 13.2:1, IL: 6% (*w*/*w*)	NA Catalyst can be reused	[102]
Chicken lard and karanja oil Solvent: protic deep eutectic from benzyl trimethyl ammonium chloride (BMC) and various organic acids	140–240 W, 10–30 min M:O 1:1–6:1 Solvent: 1–10% (*w*/*w*) Enzyme: 5% (*w*/*w*)	240 W, 30 min Met: Oil 6:1 Solvent: 8% (*w*/*w*) CL: 96.4% KO: 88.6% Enzyme: 5% (*w*/*w*) CL: 91% KO: 91.2%	For pDes treatment CL: 43.1 MJ/kg KO: 35.8 MJ/kg For enzyme catalyst CL: 39.4 MJ/kg KO: 24.7 MJ/kg	[103]
Palm oil and methanol transesterification Catalyst: imidazolium IL, [HSO_3_-Bmim] H_2_SO_4_	60–120 W 1–8 h MR of M:O 3–18 IL 4–14%	98.9% of biodiesel yield 6.43 h, 168 W, M:O 11, IL: 9.2%, at 108 °C	Save up to 44% compared to conventional	[105]
Algae *Nannochloropsis sp.* and methanol direct transesterification Catalyst: 1-ethyl-3-methylimmidazolium methyl sulfate, [EMIM][MeSO_4_]	MR of Algae to M 1:4–1:12 M to O 1:0.5–1:1 5–25 min Temp. 65–95 °C	42.4% of biodiesel per dried biomass 14 min, Algae to M 1:4 M to O 1:0.5 At 65 °C	NA	[106]
Algae *Nannochloropsis sp.* and methanol direct transesterification Solvent: methanol and 1-ethyl-3-methylimmidazolium methyl sulfate, [EMIM][MeSO_4_]	5–15 min Temp. 65 °C Difference type of solvent MR of M:O 6–12	36.8% of biodiesel per dried biomass, 15 min, At 65 °C, A:M = 1:4 M:IL = 1:0.5	NA	[107]
Primary sewage sludge and methanol Solvent: tetrakis(hydroxymethyl) phosphonium chloride, [P(CH_2_OH)_4_]CL Catalyst: 1v% of sulfuric acid	MR of sludge: IL 1:5–1:20 (g/TS: cm^3^/IL) 25–100 °C	23.4% (dried sludge) and 27.6% (raw sludge) lipid 17% (dried sludge) and 19.8% (raw sludge) biodiesel 3 h extraction, 1:5 (sludge: IL) 100 °C	Energy required to extract 1 kg of lipids was estimated to be 238 MJ/kg_lipid_	[108]
Jatropha oil and methanol transesterification Waste cooking oil Soy oil Catalyst: 4-allyl-4-methylmorpholin-4-ium bromine, [MorMeA][Br] and Sodium hydroxide, NaOH	3–8 min MR of M:O 6–12 NaOH: 0.5–1.5% IL: 0.5–1.5% Temp. 40–90 °C	98.5% 89.1% 99.4% in 6 min M:O is 9:1 1 wt.% IL + 0.75 wt.% NaOH At 70 °C	0.254 kWh 23 times lower than conventional	[109,110]

MR: Molar ratio. M: Methanol. O: Oil. IL: ionic liquid. NA: not available.

**Table 7:** Summary of microwave-combined technology.

Type of Combined Technology	Advantages	Disadvantages
Microwave and acoustic cavitation	Improved heat and mass transfer [111] Short transesterification reaction time and high yields [77] Minimize the energy consumption and the use of hazardous chemicals [112]	Industrial scale-up difficulty [113] Inefficient ultrasound generation and high operating costs [113]
Microwave and supercritical/ subcritical	Reduction of cost for catalyst Eliminate separation and purification steps [86]	Large excess of methanol/ ethanol [86] Huge energy consumption for a reaction at supercritical condition at industry scale [94]
Microwave and vacuum	Low boiling point of the by-product, easy purification [58,96] Promotes the diffusion of reagents into the substance, increasing the contact area and accelerating the reaction [97]	High vacuum instrument consumes high energy [114]
Microwave and ionic solvent	Simplify processing step [115] Can be reused and recycle [105,106,115] Reduce amount of wastewater [115]	Expensive ionic solvent [108] Costly for traditional recovery and purification method [115] Insufficient information for whole life cycle assessment [115]

## Data Availability

All relevant and required data were extracted from included articles and were all duly cited. Please contact the corresponding author or the first author for further clarifications.
